# Adaptation, aging, and genomic information

**DOI:** 10.18632/aging.100053

**Published:** 2009-05-21

**Authors:** Michael R. Rose

**Affiliations:** Department of Ecology and Evolutionary Biology, University of California, Irvine, CA 92697-2525, USA

**Keywords:** Aging, adaptation, forces of natural selection, Hamiltonian research; experimental evolution

## Abstract

Aging is not simply an accumulation of damage or inappropriate higher-order signaling, though it does secondarily involve both of these subsidiary mechanisms. Rather, aging occurs because of the extensive absence of adaptive genomic information required for survival to, and function at, later adult ages, due to the declining forces of natural selection during adult life. This absence of information then secondarily leads to misallocations and damage at every level of biological organization. But the primary problem is a failure of adaptation at later ages. Contemporary proposals concerning means by which human aging can be ended or cured which are based on simple signaling or damage theories will thus reliably fail. Strategies based on reverse-engineering age-extended adaptation using experimental evolution and genomics offer the prospect of systematically greater success.

## Introduction

If I may begin on a personal note, it has taken me more than 30 years to understand why the evolutionary biology of aging is not adequately understood by the majority of gerontologists, to say nothing of the overwhelming majority of the non-biologists who are interested in the problem of aging. It's not that we evolutionists haven't tried. In my own case, I have repeatedly tried to convey the evolutionary approach to the gerontological community, in both books and articles, across a variety of levels and venues. During the last three decades, I have developed some understanding of the great conceptual gulf between gerontology and evolutionary biology. In the last few years, as the evolutionary biology of aging has been revolutionized by a deeper understanding of the Hamiltonian foundations of aging and late life [1], I have been particularly pessimistic about bridging this gulf, because the evolutionary theory and experimentation in this area have become yet more abstruse, compared to the intuitively more digestible evolutionary biology of aging that we had circa 1990 [2].

But I think I have now found a possible solution to this communication problem, a solution that might bridge the gulf between evolutionary biologists and the gerontological community. This solution is the concern of the present article; ideally it will help resolve a "two cultures" problem that has afflicted, and indeed impaired, gerontology for more than a century. In addition, as a secondary issue, I believe that the explanatory breakthrough that I offer here should help the gerontological community see the cogency of the intuitive disquiet that many of them feel about recent attempts to "end" or "cure" aging, such as the SENS proposal of de Grey [3]. I will begin by stating my central thesis baldly. The rest of the article will then attempt to explain and unpack this thesis for those who aren't evolutionary biologists.

Species that have distinguishable adults enjoy early adult health because of adaptations built by natural selection acting over entire genomes, adaptations that are the cumulative product of many millions of years of evolution building vast libraries of adaptive genomic information. These adaptations are fine-tuned according to the recent evolutionary histories of populations of these species, where this fine-tuning involves thousands of nucleotide base-pairs distributed across entire genomes with effects that are typically both pleiotropic and epistatic across component adaptive functions. When forces of natural selection acting on the physiological processes that underlie adaptation progressively weaken at later adult ages, as they must in all organisms with ovigerous reproduction according to Hamiltonian theory [1,4], there is a *reduction in the adaptive genomic information *required for survival and reproduction at those ages. This reduction in genomic information then leads to innumerable and pervasive failures of physiological tuning at later ages, including dysfunctional allocative signaling and cumulative cell damage, among many other types of adaptive failure. Since adaptation involves the careful tuning of functions at *every* level, from molecule to cell to tissue to organ to overall bodily integration, the failure of adaptation necessarily will take place at *all *of these levels as well. This is what the absence of the required adaptive genomic information necessarily produces at later adult ages.

Nonetheless, it is easy for evolution by natural selection to build the adaptive genomic information-base required to sustain healthspan radically. There are no absolute physiological barriers to it doing so. This is dramatically revealed by the following: (i) the non-aging species in which the forces of natural selection never fall; (ii) the non-aging germ lines of all species that are not undergoing evolutionary genomic meltdown; and (iii) the ease with which experimental evolution can slow aging when the forces of natural selection are manipulated in the laboratory. More accurately, these statements are respectively better rendered as follows: (i) adaptation is maintained at all ages in species in which the forces of natural selection never fall; (ii) germ lines are indefinitely sustainable in species that are not undergoing evolutionary genomic meltdown; and (iii) the genomic information required for prolonged age-specific adaptation is easily produced by outbred laboratory populations in which the forces of natural selection are artificially sustained at high levels at later ages.

I will now set about explaining these ideas step-by-step, in effect supplying a brief tutorial in adaptational genomics with particular relevance to the problem of aging.

### Adaptation and the genome

The chief way in which biologists first learn about the genetic basis of evolutionary change is with simple examples like the substitution of melanic alleles at pigmentation loci in the case of Lepidopteran industrial melanism. I have used this pedagogical cliché in an evolution textbook myself [5]. This example makes it seem as if natural selection characteristically works by targeting a single gene, with evolution achieving a simple substitution of one allele for another. Another classic textbook example of natural selection is the sickle-cell hemoglobin polymorphism, in which heterozygotes for the sickling and normal alleles have both resistance to malaria and reasonable erythrocyte structure, most of the time. Again, this simple example suggests the action of natural selection to solve adaptive problems using alleles of large effect at a single locus.

Unfortunately, these examples are wholly misleading. In most cases of a strong phenotypic response to selection, in either nature or the laboratory, there are a large number of loci involved. It was precisely this problem that was at the root of many of Darwinism's problems incorporating Mendelian genetics, before R.A. Fisher created quantitative genetics in 1918 [6]. Quantitative genetics is the tool that evolutionary geneticists, and significantly plant and animal breeders as well, use to deal with the inheritance of "quantitative characters" or "complex traits." Conversely, when we have cases in which natural selection maintains genetic variation, as in the case of the alcohol dehydrogenase locus in *Drosophila melanogaster, *the selective mechanisms that underlie such polymorphism are often remarkably obscure. That is, unlike the textbook cases of industrial melanism and sickle-cell anemia, the relationship between natural selection and the genome is characteristically "many-to-many," with numerous loci and complex phenotypic effects of selection being the norm. This is why the study of the genetic basis of evolutionary change is so difficult [7,8].

In a few cases, we can find simple connections between the genome and particular phenotypes, but these are not representative. This should not be surprising. Any allelic difference that can improve three or four characters will be favored by natural selection, possibly even if it has deleterious effects on one or two other characters that have less net impact on Darwinian fitness. Alleles are selected on the basis of their average effects on the entire adaptive phenotype, as quantified by Darwinian fitness, over the range of genotypic backgrounds in which they occur.

Even though we find it easier to imagine that a gene is "for" one attribute or another, evolution by natural selection can exploit pleiotropic effects across multiple characters whenever they arise. It isn't constrained by our limited understanding, or any need to be elegant. Evolution is genomically complex. It builds adaptations by accumulating information of great genomic and functional complexity over millions of years, indeed billions of years. That is, adaptations reflect the long-term accretion of useful genomic information, information that functionally manifests itself in the fine-tuning of networks of interacting elements, both proteins and RNAs of widely varying sizes and roles. That is why superoxide dismutase proteins can be found in so many different organisms; superoxide dismutases are very useful enzymes in vast numbers of different species, in each of which they undergo further fine-tuning in order to improve their operation in the particular physiological networks that underlie the survival and reproduction of members of that particular species.

### Aging: what happens when adaptation goes away

As Weismann and other evolutionary biologists have realized over the last century and more [9], when natural selection pays attention to survival to, and reproduction at, later ages it is trivial for evolution to build adaptations that will enable organisms to do both of these things. Thus it is easy for evolutionary biologists to deliberately produce organisms with slowed or postponed aging, as our publications have shown since 1980 [1,4]. All we have to do is extend the period during which the forces of natural selection act with full force. Furthermore, it is clear that this entails genetic and functional changes involving many loci [10].

Conversely, what gerontology normally does is characterize the breakdown of age-specific adaptation, even though most gerontologists don't think of themselves as evolutionary biologists. Failing to take the adaptational genome-wide foundations of aging can lead to numerous problems of experimental design and interpretation. For example, gerontologists may worry about whether or not they should be studying the maintenance of fertility and other adaptive functions, such as competitive ability, that are of no direct relevance to the survival of isolated individuals. Is, for example, a sterile fruit fly or a castrated salmon that lives much longer actually an example of slowed aging? What about a dwarf mouse that can only be kept alive with a "companion nurse" mouse, to keep it warm? Or a nematode that lives much longer in one laboratory protocol, but which has impaired survival or competitive function under other laboratory conditions [11]? These paradoxes arise because, in studying aging, gerontologists are studying the genome-wide breakdown of adaptation. The study of adaptation over entire genomes is laden with both conceptual and experimental complexities. I now give some typical examples of relevance to the study of aging. These complexities illustrate the extent to which standard gerontological experiments are entangled with issues from evolutionary biology.

(1) Inbreeding depression tends to degrade adaptation at every age. This makes the study of heavily inbred laboratory animals particularly inappropriate for research on aging, because aging is a phenomenon which hinges on the loss of adaptation with adult age. Inbred animals will show impairments of adaptation at every age, obscuring the essential feature of aging as a period of progressively impaired adaptation that follows a period of adequate adaptation.

(2) Genotype-by-environment interactions arise when organisms are assayed in different environments, particularly environments that are evolutionarily novel [12]. In the context of aging research, this can make genetic effects on longevity difficult to reproduce when protocols are changed [13]. More profoundly, studying the loss of adaptation in an organism that has not already adapted to the laboratory setting employed in an experiment will obscure what is going on in its aging, functionally, genetically, and physiologically.

(3) The adaptive "costs of reproduction" underlie the well-known antagonistic pleiotropy mechanism for the evolution of aging [14]. The costs of reproduction can also supply a ready way to generate extended lifespan, simply by reducing fecundity [15]. Thus evolutionary biologists expect that experimental manipulations which attenuate reproduction will often increase longevity, but because this involves trading one adaptation for another, we do not regard the resulting demography as a case of slowed or postponed aging.

(4) Significantly, these last two genetic mechanisms can interact, genotype-by-environment interaction making it difficult to detect genetic trade-offs, even when gerontologists are trying to find them [11]. This difficulty will be still greater when gerontologists are not particularly keen to find evidence for such trade-offs.

(5) "Longevity mutants" will characteristically suffer from adverse pleiotropic effects in at least some environments, because generally superior "longevity assurance" mutants will be favored in nature. Vastly more mutations will have been generated in the evolution of any species than we will ever produce in our laboratories in the comparatively short-term and small-scale mutation screens that we can perform. Generally beneficent alleles should have already been fixed in the course of evolution by natural selection, since they would be key adaptive substitutions.

### Aging is not a disease

There are those who seek to have the NIH declare that aging is "a disease," so that the FDA can review applications for pharmaceutical status of agents that claim to "cure this disease." There is no question that individuals who suffer from either juvenile or adult onset progerias, be they Hutchinson-Gilford's progeria or Werner's syndrome, are indeed suffering from diseases. They have well-defined spectra of pathologies that are due to single genetic differences. Pharmaceuticals or somatic gene engineering interventions can usefully target such genetic diseases, and are worthy of FDA approval if they work well.

What the rest of us have is a failure of natural selection to build the adaptations required for our continued survival and reproduction. As such, while there is a well-defined underlying evolutionary cause, the spectrum of aging pathologies that we suffer are multifarious and the loci that are responsible for them are numerous and sometimes even diffuse in their responsibility, just like the loci that are responsible for most of our adaptations.

Aging is not a well-defined disease. If such an "aging disease" terminology were adopted, it would be just as reasonable to say that wolves suffer from the disease of lacking adaptations to survive in human habitats, unlike dogs, which do have such adaptations. What then is the cure for the wolf disease?

### Nor is aging merely cumulative damage

Part-whole confusions are commonplace targets of logical training. Aging can result in cumulative damage without itself being nothing more than cumulative damage, unless the term "damage" is inappropriately broadened to mean anything that is involved in aging. This would lead to as many confusions as redefining the term "disease" to include aging.

Yet the field of gerontology has assumed that some type(s) of inexorable damage or cumulative disruption is the entire source of aging, from its inception with Aristotle to the free-radical theory of aging to the recently developed rationale for SENS [3,16]. This assumption began to unravel thanks to the work of Carey et al. [17] and Curtsinger et al. [18], which first showed the demographic cessation of aging. These trailblazing publications set evolutionary biologists to work re-evaluating their interpretation of the functional impact of the plateaus in the forces of natural selection which follow their decline [19,20]. How can the declines with age in survival probability and fecundity cease, if aging is due to cumulative damage? They can't. But they can in straightforward evolutionary models [19,21].

The conundrum of aging coming to a stop, and its resolution using evolutionary theory [19,21] and experimental evolution [e.g. 22], reveals yet again how the foundations of gerontology actually lie within evolutionary biology. Puzzles like whether or not aging can possibly come to an end late in life are readily resolved using the research tools of evolutionary biology, both theoretical and experimental.

### The attempt to "End Aging"

But things become still worse when we turn to proposals for "ending aging," "curing the aging disease," and other recent aspirational infirmities of the anti-aging movement. As I will now argue, it is particularly when we turn to these hopes that it becomes obvious that gerontology needs evolutionary foundations and tools.

Let us start with the advocates of hormone supplementation as a cure for "the aging disease." There is at least some bare evolutionary credibility behind such proposals, in that hormonal manipulations are heavily implicated in the beneficial responses to castration in both animal and plant species [23]. Hormones are indeed the master controls of life-history, and changing their levels demonstrably has pervasive functional effects [24]. But the problem is that antagonistic pleiotropy is the likely mechanism behind the benefits that have been realized from hormonal manipulation. Because reproduction is costly, limiting it hormonally should sometimes produce longevity benefits.

Ironically, the most widely used hormone interventions in "anti-aging medicine" involve hormone supplementation with growth hormone or sex steroids, which experimental data and evolutionary theory both suggest will decrease lifespan and later-life somatic functions, thanks to increased physiological investment in costly functions related to reproduction. This couldn't be a more ill-founded therapeutic strategy, from the standpoint of evolutionary theory.

Then we have those pharmaceutical strategies that are based on emulating the pathways implicated in the response of lifespan to dietary restriction, particularly sirtuin-targeting agents like resveratrol [e.g. 25]. Again, like hormone manipulation, these pathways are heavily bound up with the regulation of reproduction, making the curtailment of the cost of reproduction the most likely mechanism by which the beneficial effects of emulating dietary restriction are achieved [cf. 26]. This is a strategy in which longevity is increased by metabolic refrigeration, pseudo-hibernation, or curtailing functions [11]. From the standpoint of evolutionary biology, this is, again, not an extension of the period of adaptation. It is instead trading one set of adaptations off against another. Most people do not regard curtailing their metabolism, cognition, affective stability or reproductive functions as a useful approach to the problem of aging. Nonetheless, some are willing to trade-off some of their adaptive functions for an increased lifespan, and for them this "anti-aging" strategy will have its attractions.

Finally, we have SENS [3]. Taking "Strategies for Engineering Negligible Senescence" literally as worded, it is impossible to object to, at least as a technological ambition. The problems arise with the conceit that aging is due entirely to seven types of cellular or molecular damage. For those well-trained in medical pathology, this assertion flies directly in the face of the remarkably diverse ways in which the aging human body manages to get things wrong as it becomes older. Aging involves derelictions of function at many different levels of biological organization, in strikingly heterogeneous ways across different types of tissue and organ, as a reasonable reading of the aging literature, medical or comparative, would show an open-minded reader [vid. 23]. And this is just as evolutionary biologists have long expected.

Aging is not due to the progressive breakdown of a complex biochemical machine due to accretions of damage afflicting an entity that could otherwise continue functioning indefinitely. The fact that it is well within the capacity of evolution by natural selection to produce organisms that don't age shows that there is something wrong with this assumption. Instead, evolution by natural selection *does not bother *building ovigerous organisms that can live indefinitely, because the genomically-complex and information-laden adaptations required for indefinite survival of such adult somata are not favored by natural selection. This results in the *absence* of the substantial amount of genomic information that is required to sustain life indefinitely. It isn't damaged information, any more than the aging body merely suffers damage during aging and nothing else. The adaptive genomic information required for indefinite survival simply hasn't been produced by evolution.

### Extending healthspan requires extending adaptation

To evolutionary biologists, then, the problem of extending healthspan is how to produce the adaptations required to sustain high levels of function at later ages. Evolutionary biologists have known since the 1950's that lifespan can be increased fairly predictably by curtailing reproduction and its associated physiological costs and stresses. Drugs, surgery, and diet can all have this effect. But the problem of a true extension of "a full life," or "healthspan," is more difficult, because it is nothing less than building a new set of later-age adaptations.

 This difficult problem becomes trivially easy for us to solve when we can control Hamilton's [1,4] forces of natural selection: when we tune these forces up at later ages in genetically variable populations over a number of generations, increased healthspan or, to us evolutionary biologists, prolonged adaptation is the predictable result [2,27].

It is organisms whose evolution we can't control that pose a severe technological problem of aging for evolutionists. Evidently, as both a practical and an ethical matter, we can't control human evolution. Contemporary evolutionists think of this as an impractical and morally dubious project, contrary to the eugenicists of the early 20^th^ Century.

On the view of aging presented here, extending human healthspan requires that we find means by which we can emulate what natural selection has already given us at earlier ages: a broad spectrum of adaptations that have been produced by millions of years of fine-tuning the vast amount of information stored in each mammalian genome. This is such a daunting prospect that most evolutionary biologists, who generally understand the point that the evolution of aging is founded on a failure of adaptation, regard the slowing of human aging as an essentially intractable problem. But at least some evolutionists who used to feel that way have now changed their minds.

### Evolutionary strategies for extending human adaptation to later ages

Experimental evolution and genomics are the technologies that have changed the prospects for extending human adaptation to later ages, in the eyes of some evolutionary biologists at least. I will explain how by following the historical progression by which this possibility was conceived by evolutionary biologists.

Experimental evolution is the technique that has best fostered the scientific evaluation of the evolutionary theories and mechanisms of aging [28]. Experimental evolution is an empirical approach based on setting up conditions in which replicated populations are allowed to evolve, using either *de novo* mutations [29] or standing genetic variation [30] as "fuel" for the evolutionary process, and focused selection pressures of some type as the "steering" for the evolutionary process [31]. Some of the first tests of the basic Hamiltonian evolutionary theory for aging were performed using experimental protocols in which the first age of reproduction was progressively postponed in outbred *Drosophila *populations over more than ten generations [32]. For the last 30 years, such evolution experiments have repeatedly corroborated the evolutionary theory of aging [28].

But more importantly, for the present purpose, these experiments have produced a variety of experimentally evolved populations in which adaptation has been sustained well on to later ages. That is, organisms with slowed aging have been derived from organisms with a faster rate of aging; to use the language of adaptational biology, we have been able to extend the period of adequate adaptation from early life to later life. Comparing the extended-adaptation *Drosophila* with their matched controls has allowed useful physiological and genetic analysis of the mechanistic basis of extending adaptation [27]. In particular, it was soon estimated that many genes were involved in this prolongation of *Drosophila *adaptation, implicating a large amount of genomic change in the re-tuning of the aging process [10].

In the 1980s, evolutionary biologists did not think longer-lived *Drosophila *would provide much, if any, useful guide to the specific genomic foundations of aging in humans, because of then-prevailing expectations among evolutionary biologists that there would be wide divergence of genomes among phylogenetically distant species [vid. [33]]. (In this respect, the prejudices of many molecular biologists were more accurate than those of mainstream evolutionary biologists before the advent of genomics.) Thus it was proposed that progress in finding the right genomic and physiological information for postponing human aging required the development of mice with selectively postponed aging [34]. Unfortunately, while it was shown that the same evolutionary methods as those used with *Drosophila *alsoworked in a small-scale mouse experiment [35], the level of replication used in that experiment was not sufficient to make these mice promising material for genetic or physiological analysis. Despite repeated attempts to produce a consensus that the creation of properly replicated populations of mice with evolutionarily extended adaptation would benefit gerontological research, the project was never attempted [36]. This seemed to preclude any reasonable prospect for evolutionary biology contributing to the amelioration of human aging.

The advent of whole-genome technologies, or "genomics," circa the year 2000 changed the situation substantially. Most importantly, it was realized that there was far more orthology among the genes of metazoan genomes than had been anticipated by evolutionary biologists, particularly within the segmented bilaterian group, which includes both insects and mammals. Genome-wide studies comparing the loci involved in *Drosophila *aging with the entire human genome give estimates of orthology that are quite high, well over 80% [37], suggesting the possibility of extrapolating from *Drosophila* evolutionary genomics to humans in the case of aging. This may be because the many loci which must evolve in order to sustain adaptation at later ages are involved in common "housekeeping" functions, such as energetic metabolism - which has been extensively implicated in the evolution of slowed aging in fruit flies [27], rather than lineage-specific loci, in most cases. This in turn leads to the possibility of developing pharmaceutical and other interventions for human aging based on research that starts with the genomic information required to sustain adaptation, and thus health, in older fruit flies [36-39].

Naturally, any such genomic short-cut to reverse-engineering the evolution of slowed aging from fruit flies to humans is fraught with potential for error. Such evolutionarily deep orthologies are sure to supply incomplete information. The originally proposed evolutionarily slowed-aging mouse remains the best model organism for a project of this kind. But creating it will be a demanding project, requiring years to produce fruitful results, and substantial resources [34,39]. For the time being, the most feasible evolutionary strategy for extending human adaptation into later years is that based on experimental evolution with model organisms like *Drosophila, *particularly because such organisms already exist and the physiological changes associated with their slowed aging have already been studied extensively.

### Conclusion: bridging the two cultures of aging research

This review evidently proposes that both the scientific foundations of gerontology and the systematic, rather than adventitious, slowing of human aging would benefit from the use of evolutionary theory and experimental evolution augmented with genomics. This is because aging is properly understood as the fading out of adaptation with adult age. As evolutionary biologists have been studying adaptation for 150 years, and because we have an extensive tool kit for such research [40], we evolutionists naturally think that progress in gerontological research would be materially accelerated by the use of evolutionary foundations and tools [2,41]. This doesn't mean gerontologists becoming evolutionary biologists, anymore than human geneticists are all population geneticists. But it does mean bridging the gaps between gerontology and evolutionary biology, just as human geneticists talk and collaborate with population geneticists.

Nor should this be construed as an argument that gerontology can do without any of the skills that are deployed so resourcefully by mainstream gerontologists. Evolutionary biologists have a long history, indeed as old as Charles Darwin's original publications, of making use of all elements of biology, both as sources of information and as sources of useful tools for their own experiments. Evolutionary research on aging has been no exception to this overall pattern.

But evolutionists are generally united in their impatience with gerontological theories that are incompatible with evolutionary theory. And we chiefly feel scorn toward "anti-aging medicine" that is based on presumptions precisely contrary to evolutionary biology, just as we feel toward creationism. We offer the formal foundation necessary for mathematically sorting out much gerontological theorizing. We can supply useful experimental strategies from our longstanding tradition of studying adaptation, and we can demonstrably produce model organisms with extended healthspan at will. We feel that gerontology can prosper if it makes use of these substantive contributions.

I hope that I have managed to explain to a few more gerontologists why they should make material use of evolutionary thinking in their theoretical and experimental research. It provides concrete foundations, not window dressings.
